# The extent of the psychological impairment of prosthodontic outpatients at a German University Hospital

**DOI:** 10.1186/1746-160X-4-23

**Published:** 2008-10-23

**Authors:** Michelle A Ommerborn, Alfons Hugger, Johannes Kruse, Jörg GK Handschel, Rita A Depprich, Ulrich Stüttgen, Stefan Zimmer, Wolfgang HM Raab

**Affiliations:** 1 Department of Operative and Preventive Dentistry and Endodontics, Heinrich-Heine-University Düsseldorf, Moorenstr. 5, 40225 Düsseldorf, Germany; 2 Department of Prosthodontics, Heinrich-Heine-University Düsseldorf, Moorenstr. 5, 40225 Düsseldorf, Germany; 3 Clinical Institute of Psychosomatic Medicine and Psychotherapy, Heinrich-Heine-University Düsseldorf, Moorenstr. 5, 40225 Düsseldorf, Germany; 4 Department of Cranio- and Maxillofacial Surgery, Heinrich-Heine-University Düsseldorf, Moorenstr. 5, 40225 Düsseldorf, Germany; 5 Department of Operative and Preventive Dentistry, University of Witten/Herdecke, Alfred-Herrhausen-Str. 50, 58448 Witten, Germany

## Abstract

**Background:**

Psychological factors are not only important in patients with temporomandibular disorders (TMDs), but also in patients suffering from tooth loss and/or in those awaiting prosthodontic care with fixed or removable dentures as several authors emphasize. The purpose of the present prospective observational study was to compare prosthodontic outpatients of the Department of Prosthodontics at the University of Duesseldorf and patients seeking care at the TMD/Orofacial Pain Outpatient Clinic (TMD/OFPOC) at the same university with respect to sociodemographic data, self-reported somatic complaints, and psychological impairment.

**Methods:**

A total of 234 patients received two self-administered questionnaires including the Symptom-Check-List. Complete data have been obtained from 65 prosthodontic outpatients and 60 patients of the TMD/OFPOC.

**Results:**

Results indicated statistically significant group differences regarding sociodemographic data and somatic complaints. Concerning the latter, in 11 of the 21 items, groups differed significantly and confirmed the absence of any mixing between the two outpatient clinics. Although the evaluation of psychological impairment revealed no significant group differences, in 21.9% of the prosthodontic outpatients and in 22.0% of the patients from the TMD/OFPOC, the extent of the determined psychological impairment was similar to that of psychotherapeutic outpatients; in 9.4% and 8.5% it was similar to that of psychotherapeutic inpatients, respectively.

**Conclusion:**

Within the limitations of this study, in approximately one third of the evaluated patients of both the prosthodontic outpatient clinic and the TMD/OFPOC the psychological impairment reached values comparable to those of psychotherapeutic outpatients and psychotherapeutic inpatients. Therefore, the present findings emphasize the need to intensify the integration of psychosomatic aspects into dentistry and, in particular, to add psychological considerations to future German education plans.

## Background

While the prosthodontic management of patients with fixed partial dentures, removable partial dentures, and complete dentures represents a routine situation in daily dental practice, a lot of patients have difficulties adapting to the sensation of a foreign body [1]. This may be due to the fact that the oral environment is an extremely sensitive region [2] which can be affected by both physical and psychological stressors [1]. To give an example for physical impairment as a result of prosthodontic management: wearing removable partial or complete dentures often impairs masticatory function and speech transiently [2,3]. Further physical problems such as soreness of denture-supporting tissues, tooth movement, carious lesions at the abutment teeth [4], and temporomandibular disorders [1,5] have been documented. Apart from these physical reasons that may potentially cause problems following prosthodontic management, several authors also underline the role of psychological factors in the process of accepting removable dentures and adapting to them [3,5-7].

In general, psychological response to tooth loss and denture wearing may be influenced by the patient's personality and, thus, may sometimes depend less on the technical quality of the dentures [3]. For instance, one investigator found somatization to be a factor in general dental practice per se [8], whereas some researchers assumed subjective body complaints to be an indicator of somatization particularly in elderly patients [9]. Hence, a clear association between age and irreversible complications during prosthetic rehabilitation has been found [10] and, eventually, many of these patients are referred to specialized dental clinics, such as prosthodontic outpatient clinics at university hospitals.

Another group of patients that is typically referred to special university-based care clinics consists of patients suffering from temporomandibular disorders (TMDs). Although various factors, such as anatomy, trauma, pathophysiology, and psychology have been suggested to be involved in the development of TMD, the etiology has not definitely been clarified so far [11-14]. According to the literature, psychosocial factors have become a field of increasing interest. For instance, several studies have clearly indicated that compared to matched controls TMD patients had higher levels of psychological distress, including anxiety, somatization, and depression [15-17].

Considering this and the fact that typically both prosthodontic outpatient clinics and TMD/orofacial pain outpatient clinics are university-based care centers, patients in these institutions were not really found to be representative and thus cannot easily be compared with patients from a general dental practice. In particular, the extent of psychological impairment of patients seeking care at a prosthodontic outpatient clinic (POC) was regarded to be more obvious than in those patients that usually turn to their general dental practitioner. Hence, a comparison of patients of two different outpatient clinics appears to be more appropriate for the evaluation. Moreover, given the fact that these outpatient clinics take part in university education programs, potentially beneficial insights resulting from such observational investigation might have a favorable effect on future education plans. Therefore, the purpose of the present prospective observational study was to compare patients of a POC of the Department of Prosthodontics at the University of Duesseldorf with patients seeking care at the TMD/Orofacial Pain Outpatient Clinic (TMD/OFPOC) at the same university. We examined sociodemographic data, self-reported somatic complaints, and psychological impairment. The hypothesis used in this investigation was that groups differ significantly regarding their sociodemographic data and self-reported somatic complaints. Conversely, it was expected that there is no difference between both groups regarding the extent of psychological impairment.

## Methods

### Sample

Following written informed consent obtained by each patient, a total of 234 subjects, 148 females and 86 males, with a mean age of 49.24 ± 15.73 years (range 15 to 89 years), participated in this investigation. One hundred patients who sought care at the POC of the Department of Prosthodontics at the University of Duesseldorf were continuously recruited. This group of patients consulted the POC predominantly for detailed advice and/or treatment planning of a new prosthodontic restoration or concerning the repair of a damaged or insufficient prosthodontic restoration, and the presence of dental pain.

In addition, 134 patients seeking care at the TMD/OFPOC at the same university for the management of TMD were also included. As derived from the individual index cards, at the first appointment each patient was previously subjected to a detailed dental functional examination of the masticatory system which had been performed by one trained dentist. According to the Research Diagnostic Criteria for Temporomandibular Disorders (RDC/TMD) [18], the most frequently determined diagnoses were: myofascial pain, anterior disc displacement, arthralgia, osteoarthritis, and osteoarthrosis. Additionally, signs of bruxism have been documented using the clinical criteria of the American Academy of Sleep Medicine for the diagnosis of sleep bruxism [19].

With respect to the observational character of the present investigation no specific inclusion or exclusion criteria were determined. Regarding the application of psychometric instruments, the only limitation for a participation in this study was that each participant was required to have good German language skills.

### Design and instruments

At the visit of either the TMD/OFPOC or the POC of the Department of Prosthodontics subjects completed two questionnaires. In total, the hand out of the questionnaires took place over a time period of seven months. Additionally, further descriptive data have been derived from their index cards. To assess sociodemographic and descriptive data (Table 1), a form had been prepared and was given to each subject.

**Table 1:** Comparison of patients from the POC vs. patients from the TMD/OFPOC regarding sociodemographic and descriptive data (percentages shown in parentheses)

	Patients from the POC (n = 65)	Patients from the TMD/OFPOC (n = 60)	p
Age (years)	53.03 ± 14.09	43.45 ± 14.01	***^a^
Range (years)	26 to 81	17 to 75	
Gender	31 F; 34 M	49 F; 11 M	***^b^
Number of children	1.22 ± 1.08	1.02 ± 1.08	n. s.^c^
Education (%)			n. s.^b^
10 years of school	48 (73.8)	38 (63.3)	
12 years of school	7 (10.8)	9 (15.0)	
13 years of school	10 (15.4)	12 (20.0)	
No graduation	0 (0)	1 (1.7)	
Marital status			n. s.^b^
Single	14 (21.5)	16 (26.7)	
Married	40 (61.5)	31 (51.7)	
Divorced	5 (7.7)	8 (13.3)	
Widowed	4 (6.2)	2 (3.3)	
Re-married	2 (3.1)	3 (5.0)	
Residence (1 missing)			n. s.^b^
Duesseldorf	39 (60.9)	26 (43.3)	
Up to 25 km outside of Duesseldorf	10 (15.6)	8 (13.3)	
More than 25 km outside of Duesseldorf	15 (23.4)	26 (43.3)	
Duration of complaints (months) (3 missing)	32.81 ± 90.6	43.50 ± 80.41	***^c^
Type of referral (8 missing)			**^b^
Referred by general dentist	16 (26.7)	30 (52.6)	
Without referral	34 (56.7)	14 (24.6)	
Miscellaneous	10 (16.7)	13 (22.8)	

n. s. = not significant; * = p < 0.05; ** = p < 0.01; *** = p < 0.001

^a ^Two-sample, two-tailed *t *test; data are presented as means ± standard deviations (SD)

^b ^Chi-square test

^c ^Mann-Whitney *U *test; data are presented as means ± standard deviations (SD)

For the assessment of individual somatic complaints each participant received a self-administered questionnaire to be completed by each individual. As shown in Table 2, it consists of 21 items assessing temporomandibular symptoms, dental complaints and details which have been frequently reported by patients from the TMD/OFPOC.

**Table 2:** Frequency distribution of several somatic complaints reported by patients from the POC and patients from the TMD/OFPOC (percentages shown in parentheses)

	Patients from the POC (n = 65)	Patients from the TMD/OFPOC (n = 60)	p
Joint clicking	13 (20.0)	46 (76.7)	***
Other TMJ sounds	3 (4.6)	14 (23.3)	**
Impaired mouth opening	8 (12.3)	36 (60.0)	***
Impaired mouth closing	7 (10.8)	13 (21.7)	n. s.
Impaired chewing	11 (16.9)	36 (60.0)	***
Masticatory muscle fatigue	5 (7.7)	19 (31.7)	**
Masticatory muscle stiffness	4 (6.2)	20 (33.3)	***
Extensive prosthodontic management prior to consultation	48 (73.8)	33 (55.0)	*
Extensive orthodontic management prior to consultation	16 (24.6)	20 (33.3)	n. s.
Extensive surgical management prior to consultation	21 (32.3)	19 (31.7)	n. s.
Extensive occlusal adjustment prior to consultation	22 (33.8)	18 (30.0)	n. s.
Presence of habits	4 (6.2)	9 (15.0)	n. s.
Nail biting	2 (3.1)	1 (1.7)	n. s.
Lip biting	2 (3.1)	8 (13.3)	*
Smoking	13 (20.0)	10 (16.7)	n. s.
Tooth hypersensitivity	23 (35.4)	22 (36.7)	n. s.
Disturbance of a single tooth	8 (12.3)	14 (23.3)	n. s.
Pain upon wide mouth opening	6 (9.2)	37 (61.7)	***
Mouth dryness/burning mouth	17 (26.2)	21 (35.0)	n. s.
Need of a specific position to correctly close the mouth	9 (13.8)	26 (43.3)	***
Self-assessment of treatment need	29 (44.6)	54 (90.0)	***

For group comparisons Chi-square test was applied: n. s. = not significant; * = p < 0.05; ** = p < 0.01; *** = p < 0.001

The Revised Symptom Checklist (SCL-90-R) is a worldwide used psychometric instrument with a high internal consistency and test-retest reliability [20-22] and in the present study it was applied in its standardized German version [23,24]. It consists of 90 items and determines the subjectively rated psychological distress that has been obtained during the past seven days. Respondents rated each item on a 5-point Likert scale (from "not at all" = 0 to "extremely" = 4). These 90 items were summarized to nine subscales which measure different psychological symptoms (somatization, obsessive-compulsive, interpersonal sensitivity, depression, anxiety, hostility, phobic anxiety, paranoid ideation, and psychoticism). As a total score of psychological impairment, the Global Severity Index (GSI) was also applied.

In addition, to estimate the clinical relevance of the obtained results, the distribution of the GSI values of both patients from the POC and patients from the TMD/OFPOC were compared with the previously determined and published GSI values of a normative sample, a sample of psychotherapeutic outpatients, and a sample of psychotherapeutic inpatients. The estimation of these reference values has been described in detail elsewhere [25]. Briefly, if the GSI values collected these three samples have been graphed, at a GSI value of 0.65 the distributional curve of the psychotherapeutic outpatient sample exceeded the curve of the normative sample. At a GSI value of 1.35 the distributional curve of the inpatient psychotherapeutic sample exceeded the curve of the psychotherapeutic outpatients. Concluding from these previously performed clinical group comparisons, in the present study the magnitude of psychological impairment of patients with a GSI value ≤ 0.65 was assigned to the field of the normative sample, that of patients with a GSI value ranging >0.65 and <1.35 to the field of psychotherapeutic outpatients, and that of patients with a GSI value ≥ 1.35 was seen in the field of psychotherapeutic inpatients, respectively.

### Missing data

Generally, subjects that had more than one missing item in the form collecting sociodemographic and descriptive data and, additionally, one or more missing item in the questionnaire to determine individual somatic complaints were excluded. According to these criteria, 109 subjects were not included in the analysis. This drop-out sample consisted of 68 females and 41 males.

Finally, the remaining 125 subjects included 65 patients from the POC and 60 patients from the TMD/OFPOC. Thus, the average response rate amounted to 53.4%. The data of two participants were not included, because the amount of missing items in the SCL-90-R compromised the calculation of the summary score. The maximum critical values of missing items still allowing the calculation of the subscales and the summary score, viz, the GSI, have been published elsewhere [24].

### Statistical analysis

The statistical analysis was performed using the statistical software "SPSS" Version 14.0. Normal distribution was tested by using the Kolmogorov-Smirnov-Test along with an assessment of histograms. The analysis of group differences between patients from the POC and patients from the TMD/OFPOC was carried out for most of the predominantly qualitative variables (e.g., gender, education, items of the somatic questionnaire) by using Chi-square test. Independent samples *t *test was only performed for age which was normally distributed as tested by Kolmogorov-Smirnov-Test. For all quantitative variables that were not normally distributed (e.g., duration of complaints, subscales of the SCL-90-R), differences were evaluated by means of the Mann-Whitney *U *test. When using the Mann-Whitney *U *test, the adequate statistical values are the mean ranks and the sum of ranks. However, to improve the comparability of the obtained results, data are presented as means and standard deviations (SD). For all statistical analyses an α-error probability of p < 0.05 was adopted as the statistically significant level.

## Results

As seen in Table 1, the comparison between patients from the POC and patients from the TMD/OFPOC revealed statistically significant differences with respect to age, gender, the duration of complaints, and the type of referral, whereas the evaluation of further sociodemographic variables, such as education, marital status, and residence, showed no significant differences between both groups (Chi-square; p = n.s. = not significant). Considering the career, patients from the TMD/OFPOC were significantly more ambitious than patients from the POC. To specify only the most relevant groups, 20% of patients from the POC and 43.3% of patients from the TMD/OFPOC were employed, 9.2% and 13.3% were in leading positions, 18.5% and 13.3% were housewives, and 30.8% and 8.3% were pensioners, respectively (Chi-square test; p < 0.05).

In 11 of the 21 items, the analysis of the distribution of somatic complaints revealed the following statistically significant differences (Table 2): patients from the TMD/OFPOC reported more frequently joint sounds in general, an impaired mandibular movement or function (such as opening, yawning, chewing), masticatory muscle discomfort, problems to correctly bring maxillary and mandibular teeth together as well as oral habits like lip biting. Moreover, these patients stated that, immediately prior to the consultation, they did not receive as much extensive prosthodontic rehabilitation as patients from the POC. Finally, patients from the TMD/OFPOC unambiguously expressed the necessity of their complaints to be treated.

Considering psychological impairment due to distress perceived during the past seven days, the comparison of the two clinical groups did not show any differences between both groups (Table 3). In neither a subscale nor in the GSI statistically significant group differences were obtained. To estimate the clinical relevance of the recorded data, the above described reference values were applied (Table 4). In consideration of these reference values, no group specific differences were observed again. Even though data failed to reach the level of statistical significance, on the basis of the present findings, the GSI values of 31.3% of patients from the POC and 30.5% of patients from the TMD/OFPOC were clearly located above the GSI values that were obtained from a normative sample. In 21.9% of patients from the POC and in 22.0% of patients from the TMD/OFPOC, the extent of the determined psychological impairment reached values that were in the range of psychotherapeutic outpatients and, moreover, in 9.4% and in 8.5% values in the range of psychotherapeutic inpatients, respectively (Chi-square; p = n.s. = not significant).

**Table 3:** Comparison of patients from the POC vs. patients from the TMD/OFPOC regarding the SCL-90-R subscales and the GSI values

	Patients from the POC (n = 64)		Patients from the TMD/OFPOC (n = 59)		
	
	Mean	SD	Mean	SD	p
Somatization	6.97	8.36	8.71	7.78	n. s.
Obsessive-compulsive	6.08	6.62	6.51	5.70	n. s.
Interpersonal sensitivity	4.91	6.57	4.07	4.63	n. s.
Depression	7.81	9.53	7.14	7.5	n. s.
Anxiety	4.67	7.52	4.68	5.02	n. s.
Hostility	2.69	4.16	2.36	2.63	n. s.
Phobic anxiety	2.30	5.18	1.19	2.48	n. s.
Paranoid ideation	3.59	4.51	2.98	3.71	n. s.
Psychoticism	3.44	4.99	3.25	4.57	n. s.
GSI	0.517	0.621	0.496	0.434	n. s.

For group comparisons the Mann-Whitney *U *test was applied: n. s. = not significant; * = p < 0.05; ** = p < 0.01; *** = p < 0.001

**Table 4:** Frequency distribution of the GSI values obtained for patients from the POC and patients from the TMD/OFPOC in consideration of the clinical reference values (percentages shown in parentheses)

	Patients from the POC (n = 64)	Patients from the TMD/OFPOC (n = 59)	p
GSI displayed according to the clinical groups			n.s.
GSI<=0.65 (normative sample)	44 (68.8)	41 (69.5)	
GSI>0.65 and <1.35 (psychotherapeutic outpatients)	14 (21.9)	13 (22.0)	
GSI>=1.35 (psychotherapeutic inpatients)	6 (9.4)	5 (8.5)	

For group comparisons Chi-square test was applied: n. s. = not significant; * = p < 0.05; ** = p < 0.01; *** = p < 0.001

## Discussion

This prospective observational clinical study compared patients from the POC at the University of Duesseldorf with patients from the TMD/OFPOC at the same university regarding sociodemographic data, self-reported somatic complaints, and psychological impairment. As a major finding, the results clearly demonstrated that the degree of psychological impairment was similarly high in both groups. Interestingly, in approximately one third of the evaluated patients of both the POC and the TMD/OFPOC the psychological impairment reached values that were located in the range of psychotherapeutic outpatients and psychotherapeutic inpatients. Indeed, this important finding supports numerous studies which revealed psychological characteristics in TMD patients [13-17,26-28]. Moreover, these results particularly stress the fact that patients from a POC represent a different group of patients showing a similar pronounced psychological impairment than do TMD patients.

Apart from dentistry, previous investigations have recorded the urban prevalence of psychiatric disorders in general practices. While depressive and anxiety disorders were detected in 15% to 25% of the patients from a general practice [29], surveys which have analyzed the entire prevalence of psychiatric disorders obtained values ranging from 21% to 52% [25,30-33]. With respect to the portion of psychologically impaired patients, it might be concluded that the present values established in the two university-based care centers are largely comparable to that derived from general practices.

Potential reasons for the magnitude of psychological impairment found in patients from a POC might be due to the reciprocal influences of tooth loss or denture wearing and psychological factors. For instance, some previous investigators suggested that the psychological response to tooth loss and denture wearing may be affected by patients' personality traits, sometimes more than by the technical quality of the dentures [3,6]. Conversely, tooth loss is reported to cause psychological impairment [3] and, moreover, previous investigators have shown that it contributes to a reduced quality of life [34].

The sociodemographic and reported somatic complaints data showed significant differences between the two groups, especially concerning age, gender, and most of the characteristic TMD symptoms. These findings were detected as a result of the sample composition. The unequivocal differentiation of the two groups by means of the reported somatic complaints, underlines the absence of any mixing between the two outpatient clinics. It might have been expected that patients suffering from tooth loss and, consequently, seeking prosthodontic management were on an average older than a group of patients from a TMD/OFPOC. Interestingly, in contrast to the patients from the TMD/OFPOC the prosthodontic outpatients revealed a comparatively balanced gender distribution. Most of these patients sought care at the POC due to apparently somatic reasons such as the improvement of mastication or speech. The reasons differ from those reported by patients from the TMD/OFPOC. Potentially, such motives do not require a greater health awareness [35,36] or specific experiences with the health care system [37] as they have been discussed to explain the pronounced female proportion in TMD patients.

Notwithstanding that groups differed with respect to age and gender, the comparison of patients of these two university-based care centers was found suitable for the following reasons: although previously several authors have discussed the interaction between psychological factors and tooth loss and/or subsequent prosthodontic management with removable dentures [3,5-7], to date, little is known of the extent of psychological impairment in patients seeking care for prosthodontic rehabilitation. Consequently, in a first exploratory approach patients collected from a POC should be investigated. Indeed, patients from a university-based care center represent a highly specific sample that may not easily be compared with patients from a general dental practice. However, if sociodemographic data of the patients seeking care at the POC of the Department of Prosthodontics at the University of Duesseldorf are compared with data derived from other German prosthodontic departments, they appear to be representative for these specific German care centers [34,38]. Regarding an adequate control group for the patients from the POC, a decision was made to investigate patients from a TMD/OFPOC at the same university due to diverse reasons. Firstly, these patients were recruited from a different university-based care center and, thus, represent a likewise highly specific sample. Secondly, many studies have underlined the contribution of psychological factors in the etiology of TMD [15-17,27,28]. This is also reflected by the currently highest standard for the diagnosis of TMD (i. e. RDC/TMD) due to the fact that it includes the assessment of specific psychological symptoms such as depression [18,39]. Therefore, TMD patients represent a group of patients who might be considered as a quite appropriate sample to compare the magnitude of psychological impairment.

The results of the present findings reproduce the typically clinical conditions and the complexity of the clinical reality [40] in German outpatient clinics. Keeping in mind that these clinics take part in university education programs and given the determined huge number of psychologically impaired patients from both the POC and the TMD/OFPOC, one might be in favor of asking to what extent these conditions find an adequate consideration in current dental education plans. In particular, in consideration of the increasing knowledge of the association between TMD and psychological factors, this demand has already been verbalized by different investigators many years ago [41-43]. Therefore, further investigations are required to substantiate the need of an increased consideration of psychological aspects in future dentistry.

## Conclusion

Within the limitations of this study, in approximately one third of the evaluated patients of both the POC and the TMD/OFPOC the psychological impairment reached values similar to those of psychotherapeutic outpatients and psychotherapeutic inpatients. Therefore, the present findings emphasize the need to intensify the integration of psychosomatic aspects into dentistry and, in particular, to add psychological considerations to future German education plans.

## List of abbreviations used

(POC): Prosthodontic Outpatient Clinic; (TMD/OFPOC): TMD/Orofacial Pain Outpatient Clinic.

## Competing interests

The authors declare that they have no competing interests.

## Authors' contributions

AH, US, and JK conceived the study design, MAO performed the statistical data analysis and wrote the manuscript, JGKH, RAD, SZ, and WHMR participated in the early preparation of the manuscript and contributed to write the revised version of the article. All authors read and approved the final manuscript.
